# RRx-001, an epigenetic-based radio- and chemosensitizer, has vascular normalizing effects on SCCVII and U87 tumors

**DOI:** 10.1186/s13148-016-0220-7

**Published:** 2016-05-11

**Authors:** Bryan Oronsky, Jan Scicinski, Pedro Cabrales, Andrew Minchinton

**Affiliations:** EpicentRx, Inc, 800 W El Camino Real, Suite 180, Mountain View, CA 94040 USA; Department of Bioengineering, University of California San Diego (UCSD), 9500 Gilman Dr, La Jolla, CA 92093 USA; Cabenda Pharmaceutical Research, 1060 East 10th Ave, Vancouver, BC V5T 2B5 Canada

**Keywords:** Tumor, Vascular normalization, Epigenetic agent, Radiosensitizer, Chemosensitizer, RRx-001

## Abstract

**Background:**

The tumor-specific microregional effects of the anticancer agent RRx-001, a novel epigenetic-based radio/chemosensitizer with nitrogen oxide-donating properties in phase II clinical trials, were investigated with whole tissue section quantitative immunohistological staining in mouse SCCVII and human U87 tumors.

**Results:**

SCCVII tumors exhibited regions of intermittent perfusion exemplified by co-localization of vessels with the hypoxia marker pimonidazole commonly occurring throughout the tissue. A moderate increase in perfusion (21 to 28 %) was observed after a bolus dose of the perivascular stain DiOC_7_(3), however, with the absence of an increase in tissue oxygenation. U87 tumors showed an absence of blood flow over large areas of treated tumors after dosing with RRx-001. However, these areas did not become necrotic and returned to near normal levels after 12 h. No significant change in tumor hypoxia was seen at 90 min or 12 h. For both tumor types, RRx-001 treatment resulted in the loss of perfusion in the large regions of the tumor; however, at the 12-h time point, both tumor types showed an increase in vessel perfusion but no significant decrease in hypoxia.

**Conclusions:**

These data suggest a redistribution of blood flow within the tumor for both tumor types akin to vascular normalization. Differences between the tumors were related to tumor architecture and distribution of alpha-smooth muscle actin (α-SMA). RRx-001 shows promise for short-term blood flow redistribution in tumors with a pericyte- and α-SMA-rich vasculature. Expression of α-SMA in tumor vasculature could therefore be useful for predicting tumor response to RRx-001.

## Background

The tumor microenvironment has been compared to a neighborhood [1]. In such an analogy, the “slums” represented by the poorly perfused, marginalized, necrotic center of the tumor could be contrasted with the better-differentiated “suburbia” of the periphery with a transition zone in between them. However, equating the periphery with high status and centrality with low status is oversimplified given the diversified pattern of “hot spots” of hypoxia and perfusion throughout the core of the tumor due to variations in vasculature development [2]. These “pockets of decay” in the tumor based on the mismatched forces of supply and demand have led to supply side antiangiogenic/antivascular strategies to push these hypoxic areas to the tipping point of eradication. However, efforts at eradication inevitably meet with difficulty and/or failure because their insularity in the tumor microenvironment transforms them into “fortresses” that are difficult for chemotherapy to penetrate.

The tumor microenvironment is a catch-all term to denote a “local” set of “neighborhood” conditions, which includes hypoxia status and the architecture of the vasculature. The hallmark of the microenvironment is heterogeneity with thinly divided, cheek-by-jowl zones of wealth and poverty in terms of blood flow and oxygenation. The driver of this heterogeneity is hypoxia-induced neovasculogenesis and metastatic dissemination, which arguably is secondary to the maturity of the vasculature. Two main categories of hypoxia are present in tumors: chronic hypoxia, also known as diffusion-limited or permanent hypoxia, and acute hypoxia, also known as perfusion-limited or transient hypoxia [3].

Vasodilator-unresponsive microvessels devoid of smooth muscle actin coverage are prone to intermittent collapse or compression, leading to acute or transient hypoxia. Acutely induced hypoxic stress may enhance the potential metastatic efficiency of the tumor to a greater degree than prolonged exposure to hypoxia since transiently hypoxic cells have the advantage of proximity to functional blood vessels and a higher energy status than chronically hypoxic cells [4].

Given the nocivity of acute hypoxia, in terms of its role in tumor aggressiveness, an alternative strategy to the hypoxia-exacerbating effects of antiangiogenesis is a redistribution of the blood supply from immature vessels, where blood flow is already sluggish or stagnant, to more mature blood vessels, thereby normalizing blood flow.

**Fig. 1:**
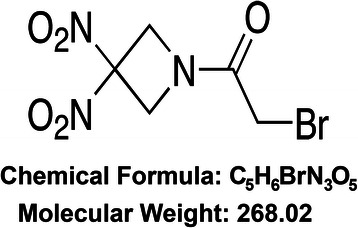
Chemical structure of RRx-001 (1-bromoacetyl-3,3-dinitroazetidine)

RRx-001, a novel investigative anticancer agent, currently under investigation in multiple phase II trials, binds to hemoglobin and influences the rheology of red blood cells, which bind to hypoxic tumor endothelium, leading to a global redistribution of blood flow [5]. In light of these pharmacokinetic and pharmacodynamic properties, we studied the vascular hemodynamics of RRx-001 and the angioarchitectural features of the murine syngeneic SCCVII tumor and the human glioma xenograft, U87, pre- and post-RRx-001 dose, to determine possible differences in vascular responsiveness and define the spatial variations in intratumoral blood flow (Fig. 1).

## Results and discussion

**Fig. 2:**
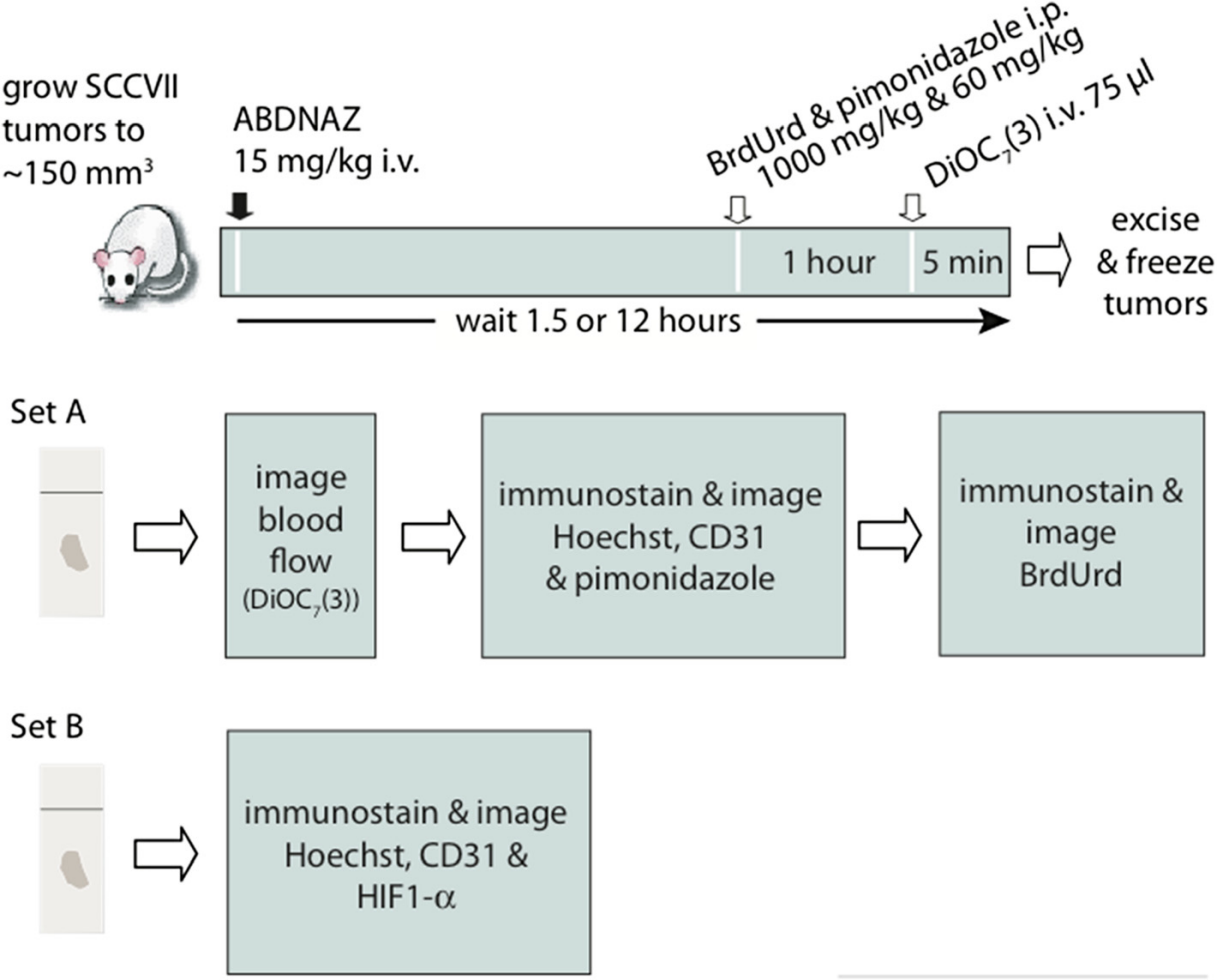
Immunohistochemistry study design. ABDNAZ = RRx-001

The microregional effects of RRx-001 on tumor blood flow and oxygenation were examined in vivo by mapping drug effects with respect to the microvascular architecture of mouse SCCVII and human U87 tumor cryosections using whole tissue mapping and multiplexed immunostaining techniques [6]. Combinations of markers including pimonidazole, CD31, DiOC_7_(3), 5-bromo-2-deoxyuridine, and HIF1-α were mapped on the tumor sections. A single dose of 15 mg/kg of RRx-001 was administered IV to C3H mice bearing SCCVII tumors and to SCID mice bearing U87 tumors. The tumors were excised 90 min and 12 h post dose then weighed and immediately frozen. Images of multiple-stained cryosections were obtained to provide microregional information on the relative position of proliferating cells, areas of hypoxia, perfusion, and vasculature after treatment (Fig. 2), and, using advanced imaging, key parameters were calculated from images of entire tumor sections.

**Fig. 3:**
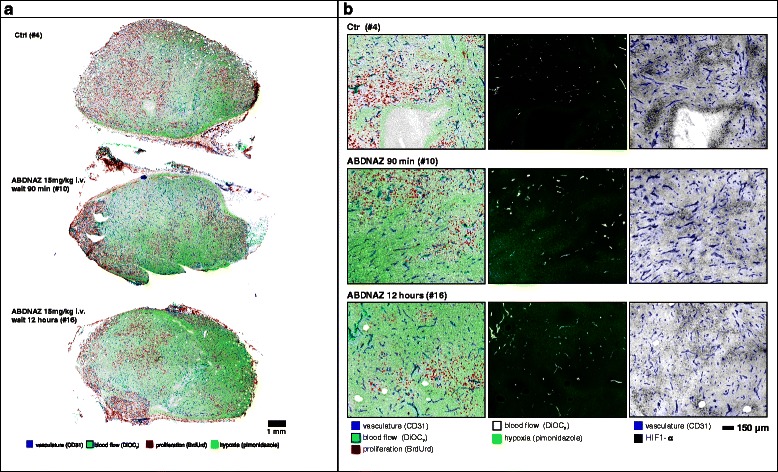
**a** Effect of RRx-001 (ABDNAZ) treatment on blood flow and tissue hypoxia in SCCVII tumors. **b** Comparison of RRx-001 (ABDNAZ) effect on tumor architecture, blood flow, hypoxia, and HIF1-α status in SCCVII tumors

**Fig. 4:**
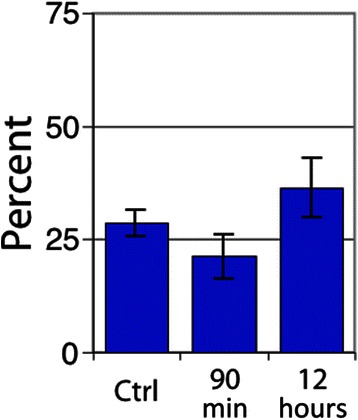
Percent of vessels positive in SCCVII tumors for the perfusion marker DiOC_7_(3). *Error bars* depict ±SD, *N* = 6 for each group, *p* < 0.05 between control and 12 h

**Fig. 5:**
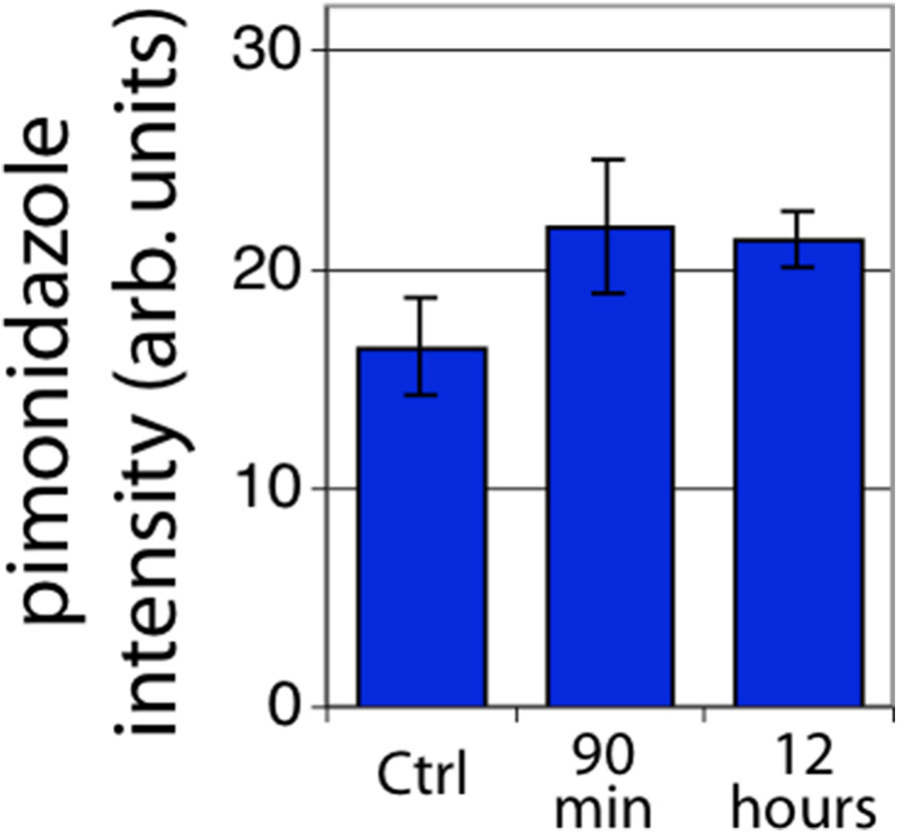
Extent of hypoxia by average tissue pimonidazole intensity staining for SCCVII tumors. *Error bars* depict ±SD, *N* = 6 for each group (not statistically significant)

**Fig. 6:**
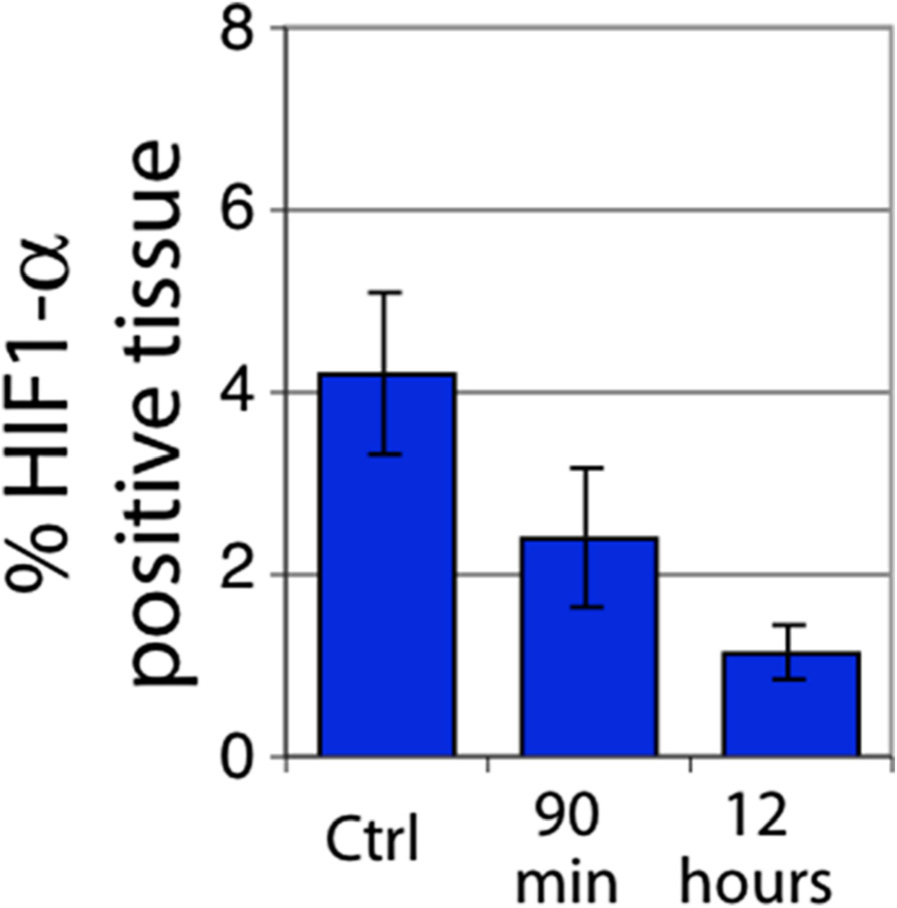
Percent tissue HIF1-α positive in SCCVII tumors. *Error bars* depict ±SD, *N* = 6 for each group (*p* < 0.05 between control and 12 h)

Vascular dysfunction was observed in a large proportion of both U87 and SCCVII tumors. SCCVII tumors exhibited regions of intermittent perfusion exemplified by co-localization of vessels with the hypoxia marker pimonidazole commonly occurring throughout the tissue (Fig. 3). Only 21 % of the vessels showed perfusion after a bolus dose of the perivascular stain DiOC_7_(3) (Fig. 4), increasing to 28 % 12 h post-RRx-001 (*p* < 0.05). Paradoxically, an increase in tissue oxygenation was not seen while modestly increased, though not statistically significant, pimonidazole binding was observed (Fig. 5). The percent of tissue that stained positive for HIF1-α was found to decrease after 90 min and 12 h (Fig. 6, *p* < 0.05 for post 12 h).

**Fig. 7:**
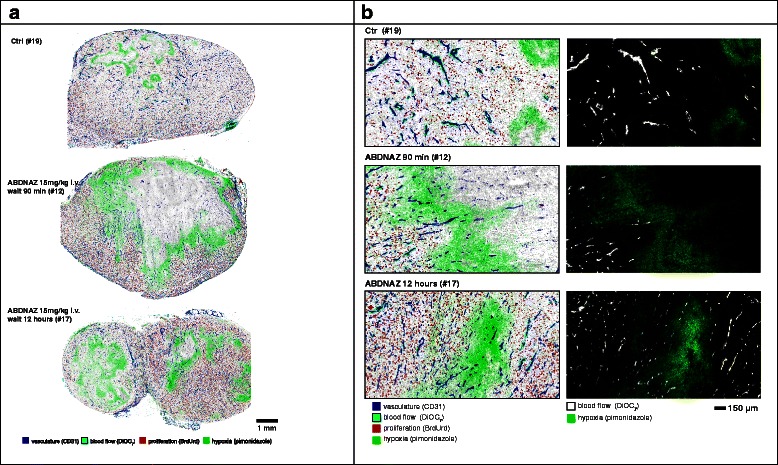
**a** Effect of RRx-001 (ABDNAZ) treatment on blood flow and tissue hypoxia in U87 tumors. **b** Comparison of RRx-001 effect on tumor architecture, blood flow, and hypoxia in U87 tumor

**Fig. 8:**
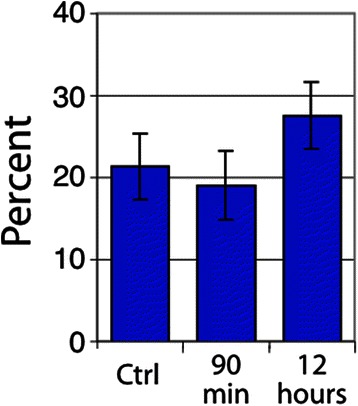
Percent of vessels positive in U87 tumors for the perfusion marker DiOC_7_(3). *Error bars* depict ± SD, *N* = 6 for each group (not statistically significant)

**Fig. 9:**
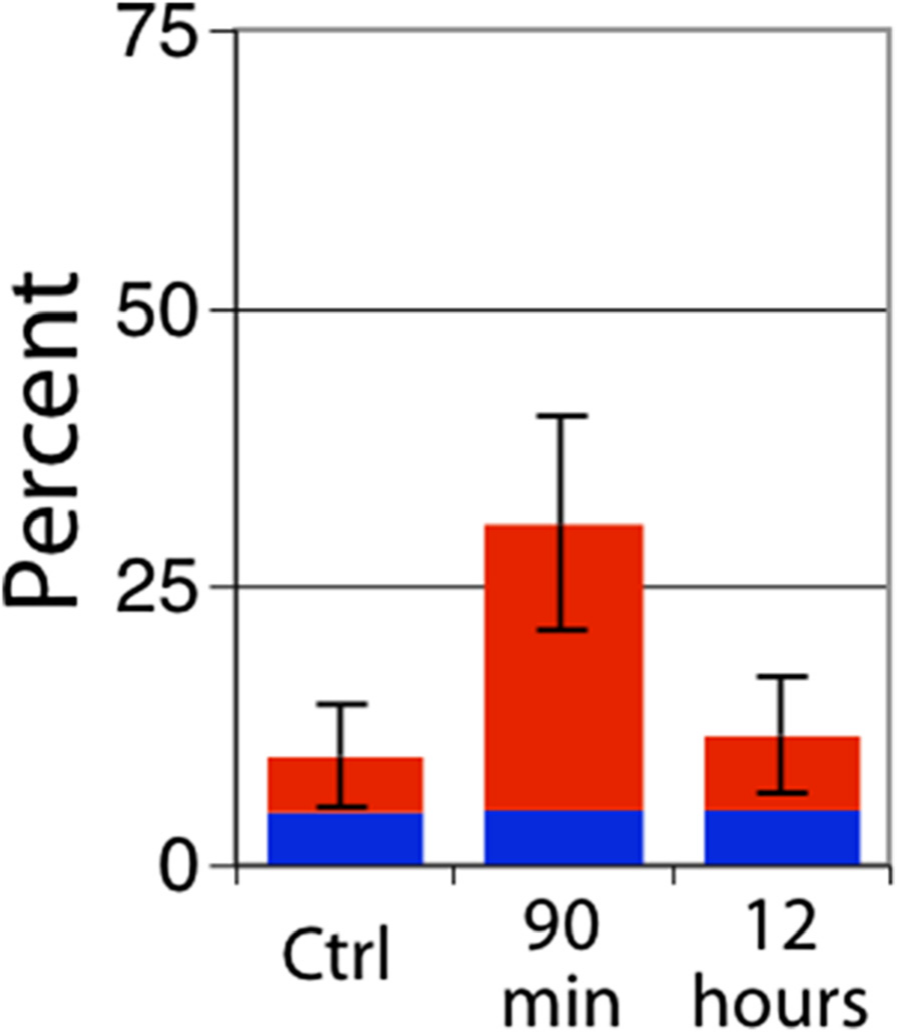
Percent tissue area exhibiting gross vascular shutdown. Regions of shutdown and necrosis were identified as a fraction of whole tissue area. Percent tumor area in U87 tumors that is necrotic (*blue square*) or unperfused (*red square*). *Error bars* depict ±SD, *N* = 6 for each group (*p* < 0.05 between control and 90 min for unperfused area, other data not significant)

**Fig. 10:**
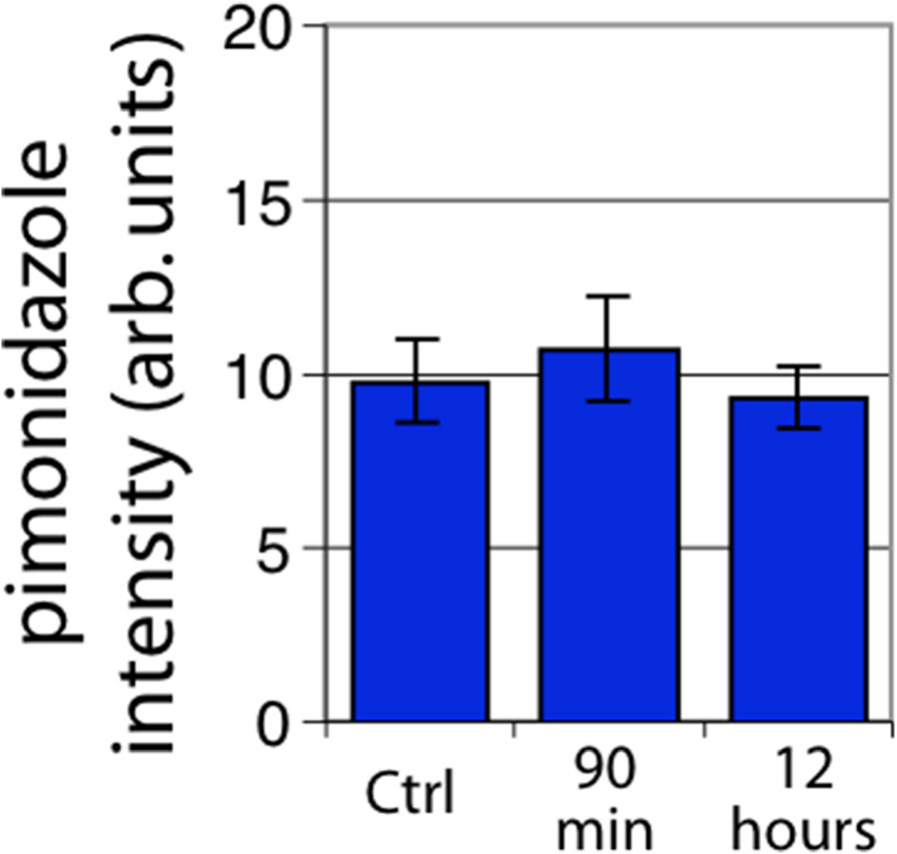
Tumor hypoxia in U87 tumors by average tissue pimonidazole intensity staining. *Error bars* depict ±SD, *N* = 6 for each group (not statistically significant)

In U87 tumors, a dramatic decrease in perfusion in the centrally located tumor vessels was observed 90 min post-RRx-001 treatment (Fig. 7). Blood flow over large areas of treated tumors was completely shut down, leaving a rim of functional vessels around the periphery of the tumor and increasing the overall level of hypoxia during that time period. The area of vascular shutdown stained positive for CD31 and tissue did not appear necrotic. Figure 8 shows the percent of vessels that were found to be positive for the perfusion marker DiOC_7_ and tracks individual vessels. In contrast, Fig. 9 quantitates “gross” tissue shutdown by area. Analysis for perfusion, though not statistically significant, indicated that less than 20 % of the vessels showed perfusion after a bolus dose of the perivascular stain DiOC_7_(3) (Fig. 8), increasing to approximately 28 % 12-h post-RRx-001. These results were consistent with findings from the SCCVII tumors (Fig. 4). The observed decrease in perfused vessels translated to an increase in unperfused tissue as shown in Fig. 9. While changes in perfusion were noted, there was no significant change in the fraction of necrosis at 90 min and 12 h, suggesting that the large, statistically significant (*p* < 0.05), unperfused regions seen at 90 min had recovered rather than become necrotic (Fig. 9). No significant change in tumor hypoxia was seen at 90 min or 12 h (Fig. 10).

For both tumor types, RRx-001 appeared to cause the loss of perfusion in the large regions of the tumor; however, at the 12-h time point, both tumor types showed an increase in vessel perfusion but no overall decrease in hypoxia: Indeed, while *overall* changes in perfusion and hypoxia are mild, local changes *within the tumor* were found to be dramatic. In contrast to previous work that demonstrated increases in RRx-001-mediated bulk tumor blood flow in SCCVII tumors by contrast-enhanced ultrasound (CEUS) [7], these data suggest a redistribution of blood flow in both tumor types while differences between the tumors were related to tumor architecture and distribution of alpha-smooth muscle actin (α-SMA).

## Conclusions

In the rhetoric of urban space, the tumor microenvironment is a heterogeneous yet integrated co-op neighborhood etched with boundaries and borderlands that intersect, overlap, collide, and coexist along lines drawn and redrawn by quirks of intricate capillary network geometries, leading to the heterogeneity of oxygen delivery and the associated oxygen gradients.

The areas with the greatest metastatic potential may not be the down-and-out “skid row” population of chronically ischemic cells but the still-viable population of acutely hypoxic cells proximate to a mature vascular network and transiently enriched with oxygen.

Antiangiogenic or antivascular strategies that inhibit the existing or the neovasculature may only serve to increase the hypoxic fraction and, thus, the metastatic efficiency of the tumor.

In contrast, the antitumor and antiproliferative effects of the novel epigenetic inhibitor, RRx-001, may be ascribed to a relative change or reorganization of blood flow within the tumor. This conclusion is supported by the macroscopic appearance of poorly perfused and hypometabolic areas in the SCCVII and U87 tumors without an increase in the overall hypoxic fraction post-RRx-001 treatment.

RRx-001-modified red blood cells selectively home to and obstruct morphologically immature and hypoxic tumor vessels, leading to a compensatory redistribution of blood flow through more mature smooth muscle pericyte-covered vessels, akin to vascular normalization. This normalization, which has been demonstrated preclinically and clinically, improves the delivery of cytotoxic chemotherapy and radiosensitizing oxygen, improving tumor control.

SCCVII tumors are characterized by a more mature angioarchitecture than U87 xenografts. The hypothesized effect of RRx-001 on SCCVII tumors is a multifocal, diffuse vasoconstriction with a uniform redistribution of blood flow in contrast to the focally vasoconstricted U87 tumors with moth-eaten cell-lucent areas.

Although contrast-enhanced ultrasound detected changes in bulk tumor blood flow in SCCVII tumors after RRx-001 administration suggesting a provascular effect [8], the data presented herein demonstrate a significant but temporary loss of perfusion with no significant concomitant change in the level of hypoxia, suggesting a redistribution of blood flow within the tumor. Differences in the local nature of the effect between the two tumor types can be attributed to tumor architecture.

The binary view of pharmacologic blood flow modulation as either pro- or antivascular should be amended to include strategies of redistribution [9]. Just like the rehabilitation of “low rent” neighborhoods characterized by overcrowding and marginalization, the redistributive strategy posits that the marginal, acutely hypoxic regions of the tumor can be revitalized or normalized instead of demolished, thereby reducing the oxygen asymmetry and the hypoxic potential for angiogenesis and metastatic dissemination.

The tumor microenvironment should not be viewed as homogenous masses of cancer cells but rather mixtures of cell populations with varying levels of heterogeneity that respond differently to different vascular modification strategies. Diagnostic imaging and histopathologic examination in the setting of vascular normalization open a window to the particular structural and biochemical characteristics of each tumor for treatment optimization and improved patient outcomes.

## Methods

### Materials

RRx-001 used in these studies was obtained from EpicentRx, Inc. RRx-001 is a cyclic nitro compound with molecular formula of C_5_H_6_BrN_3_O_5_, molecular weight of 268.02, and chemical structure depicted in Fig. 1. The synthesis and characterization of RRx-001 is reported in detail elsewhere [10].

### Ethics

The methods in this study were carried out in accordance with approved guidelines (including ARRIVE and the Canadian Council on Animal Care guidelines) and the relevant institutional review boards (IRB) at the University of California San Diego (UCSD), and British Columbia Cancer Research Centre approved the experimental protocols.

### Mice and tumors

Female C3H/Hen mice and female NOD.CB17-Prkdcscid/J mice were bred and maintained in the British Columbia Cancer Research Centre animal facility in accordance with the Canadian Council on Animal Care guidelines. The mice were allowed free access to standard laboratory rodent food and water. Tumor cells (SCCVII: 0.5 × 106 cells in 50 μL; U87: 5 × 106 cells in 50 μL) were implanted sub-cutaneously into the sacral region. The mice were administered RRx-001, 15 mg/kg IV, in a single dose when tumors reached ~150 mm^3^ as calculated from caliper measurement of three orthogonal diameters (a, b, c) using the formula volume = *π*/6(abc). Each arm of an experiment contained six mice. The mice were administered 1000 mg/kg BrdUrd i.p. and 60 mg/kg pimonidazole i.p. (Hypoxyprobe-1 Kit, Chemicon International Inc., Temecula, CA, USA) and euthanized 1 h later. Five minutes prior to carbon dioxide euthanasia and tumor harvest, the mice were administered 50 μL of 0.6 mg/mL DiOC_7_(3) in 25 % DMSO. Tumors were then excised, weighed, and immediately frozen.

### Immunohistochemistry. Group 1: DiOC_7_(3), CD31, pimonidazole, and BrdUrd

Prior to immunostaining, the slides were imaged for DiOC_7_(3) tissue fluorescence to visualize blood flow. Cryosections were fixed in a 1:1 mixture of acetone-methanol for 10 min at room temperature. Vasculature was stained using a 1:400 dilution of rat-anti-CD31 (BD 553370) and 1:200 fluorescent Alexa 488 anti-rat secondary (Invitrogen, Burlington, ON, CA). Hypoxia was detected via bound pimonidazole adducts using a 1:500 polyclonal rabbit anti-pimonidazole (Hypoxyprobe) and a 1:200 Alexa 546 anti-rabbit secondary. Cellular DNA was counterstained with Hoechst 33342. The slides were imaged for fluorescence and then transferred to distilled water for 10 min and then treated with 2 M HCl at room temperature for 1 h followed by neutralization for 5 min in 0.1 M sodium borate. The slides were then washed in distilled water and transferred to a PBS (phosphate buffered saline) bath. BrdUrd incorporated into DNA was detected using a 1:200 dilution of monoclonal mouse anti-BrdUrd (clone BU33) followed by 1:200 Alexa 647 anti-rabbit secondary.

### Immunohistochemistry. Group 2: DiOC_7_(3), CD31, and HIF1-α

Cryosections were fixed in a 1:1 mixture of acetone-methanol for 10 min at room temperature. Vasculature was stained using a 1:400 dilution of rat-anti-CD31 (BD 553370) and 1:200 fluorescent Alexa 546 anti-rat secondary (Invitrogen, Burlington, ON, CA). HIF1-α was detected using a 1:500 dilution of a rabbit anti-HIF1-α antibody (Cayman, 10006421) detected via a 1:200 Alexa 647 anti-rabbit secondary. Cellular DNA was counterstained with Hoechst 33342.

### Image acquisition

The imaging system consists of a robotic fluorescence microscope (Zeiss Axioimager Z1, Oberkochen, Germany), a cooled, monochrome CCD camera (Retiga 4000R, QImaging, Vancouver, BC, Canada), a motorized slide loader and *x*-*y* stage (Ludl Electronic Products, Hawthorne, NY, USA), and customized ImageJ software (public domain program developed at the US National Institutes of Health, available at http://rsb.info.nih.gov/ij/) running on a Macintosh computer (Apple, Cupertino, CA, USA). The system allows tiling of adjacent microscope fields of view. Using this system, images of entire tumor cryosections 1–3 cm^2^ were captured at a resolution of 0.75 μm/pixel.

### Image analysis: tumor mapping

Using NIH-ImageJ and user-supplied algorithms, images were overlaid and the areas of necrosis and staining artifacts were manually removed. On the fluorescence images, CD31-, BrdUrd-, and HIF1-α-positive regions were identified using a fixed threshold. For CD31, areas less than 9 μm^2^ in size were considered artifacts and automatically removed from the analysis. Average HIF1-α-positive staining and average pimonidazole/HIF1-α intensity were calculated from the images of the entire tumor sections following removal of the necrotic regions and tissue artifacts (folds, tears, debris, etc). The fraction of the CD31 vessels that were positive for DiOC_7_ was calculated based on a 50 % coverage by area of each CD31 object. Pimonidazole profiles in relation to tumor vasculature were calculated by measuring the distance from each point in the tissue to the nearest CD31-positive object and noting the pimonidazole intensity at that point. This data was then tabulated so as to determine the average pimonidazole staining intensity of all pixels found at each distance to a blood vessel.

### Statistical methods

Statistical analysis was conducted using GraphPad Prism (La Jolla, CA) using one-way ANOVA tests. The significance of differences between multiple groups was compared using a Bonferroni post-test analysis.
